# A Multi-Platform Optical Sensor for In Vivo and In Vitro Algae Classification

**DOI:** 10.3390/s17040912

**Published:** 2017-04-20

**Authors:** Chee-Loon Ng, Qing-Qing Chen, Jia-Jing Chua, Harold F. Hemond

**Affiliations:** 1 Singapore-MIT Alliance for Research and Technology (SMART) Centre, 1 CREATE Way, CREATE Tower, #10-01, Singapore 138602, Singapore; qingqing@smart.mit.edu (Q.-Q.C.); jiajing96@hotmail.com (J.-J.C.); 2 Massachusetts Institute of Technology, Parsons Laboratory, Room 48-425, 15 Vassar Street, Cambridge, MA 02139, USA; hfhemond@exchange.mit.edu

**Keywords:** in situ real-time algae classification, algae sensor, in vivo algae measurement, fluorescence, absorbance, optical sensor

## Abstract

Differentiation among major algal groups is important for the ecological and biogeochemical characterization of water bodies, and for practical management of water resources. It helps to discern the taxonomic groups that are beneficial to aquatic life from the organisms causing harmful algal blooms. An LED-induced fluorescence (LEDIF) instrument capable of fluorescence, absorbance, and scattering measurements; is used for in vivo and in vitro identification and quantification of four algal groups found in freshwater and marine environments. Aqueous solutions of individual and mixed dissolved biological pigments relevant to different algal groups were measured to demonstrate the LEDIF’s capabilities in measuring extracted pigments. Different genera of algae were cultivated and the cell counts of the samples were quantified with a hemacytometer and/or cellometer. Dry weight of different algae cells was also measured to determine the cell counts-to-dry weight correlations. Finally, in vivo measurements of different genus of algae at different cell concentrations and mixed algal group in the presence of humic acid were performed with the LEDIF. A field sample from a local reservoir was measured with the LEDIF and the results were verified using hemacytometer, cellometer, and microscope. The results demonstrated the LEDIF’s capabilities in classifying and quantifying different groups of live algae.

## 1. Introduction

Algae can be both beneficious and deleterious to aquatic ecosystems. Chlorophytes serves as an important food source for herbivorous marine life (e.g., fish, crustaceans, gastropods) while hepatotoxins and neurotoxins producing cyanobacteria blooms can be harmful to organisms and animals. The toxins produced by red-tide dinoflagellates can cause fish kills and shellfish contamination leading to human fatalities as well as vectorial intoxication whereby the toxins are accumulated and transported through pelagic food webs [1]. The occurrence of harmful algal blooms (HABs) in seawater can also affect the operation of seawater reverse osmosis plants due to accumulation of algal organic matter. A sudden alteration in the algal taxonomic composition may also point to a potential occurrence of water contamination, when one dominant species is replaced by another. The rapid identification of algae species is important for monitoring eutrophication and the assessment of water quality [2]. Therefore, the identification and quantification of algae in water is prerequisite for developing proactive strategies to ameliorate the negative impact of HABs.

The current assessment of the major groups of algae is both time consuming, labor intensive, and costly; which also limits the temporal and spatial resolution of the monitoring, and leads to uncertainty in the understanding of harmful taxa and the environmental conditions promoting blooming initiation, maintenance, and senescence [3]. The identification and classification of algae are usually performed using microscopy, where more recent efforts have been put on automating the system for the rapid, accurate recognition, and classification of algae using image processing techniques such as segmentation, shape feature extraction, pigment structure determination, and neural network grouping [4,5,6,7] and literatures cited therein. The system is slow, expensive, difficult to implement in the field, mostly target specific types of algae, and/or may not work well when images contain too many objects. Beutler et al., excited chlorophyll fluorescence with five distinct wavelength utilizing light-emitting diodes to differentiate spectral groups of microalgae in vivo and in situ using a submersible instrument [8]. However, looking at chlorophyll content alone may make the system prompt to erroneous estimations caused by interference from other spectral groups present in the water and matrix effects of the environment. Hu, used an ocean color index, called Floating Algae Index, to detect floating algae in open ocean environments using a moderate resolution imaging spectroradiometer [9]. The detection is fast but costly and proprietary; and is limited to the observation of algae on the surface of the water bodies in visible concentrations. It may also be susceptible to the presence of different optical interferences and matrix effects from the environment. Richardson et al., compared the Algae Online Analyser with High Performance Liquid Chromatography and Chemical Taxonomy-derived abundances and found that the former gives a good first-order approximation despite the fact that results differs statistically from the latter [10]. In addition, the determination of absolute chlorophyll a concentration was less robust and requires frequent calibration.

Ng et al. developed and field tested a multi-platform optical sensing technology utilizing multi-excitation fluorescence, broadband absorbance, and scattering to observe both spatial pattern and temporal trend of contaminants and natural substances in the water [11,12,13,14,15]. This work extends and characterizes the technology for in vivo algae classification to provide a comprehensive spectrum of water quality information.

## 2. Materials and Methods

### 2.1. Instrument

**Figure 1:**
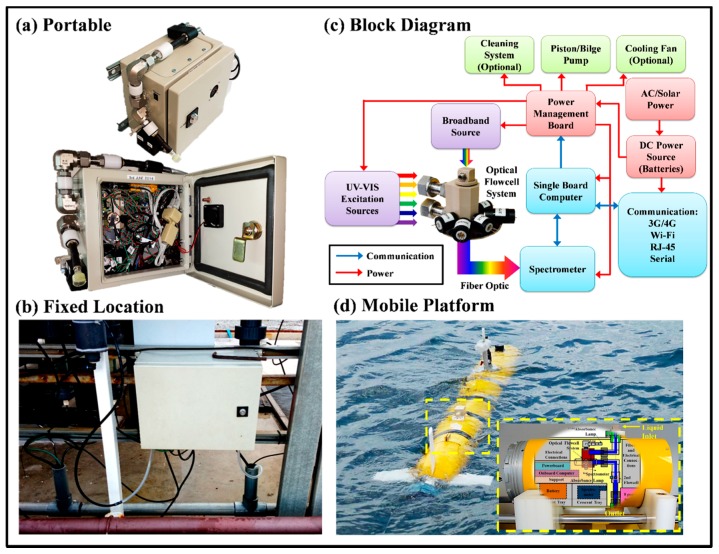
The layout and packagings of the LEDIF: (**a**) Isometric and front views of the LEDIF packaged inside a 20 × 15 × 20 cm enclosure for portable mode sensing; (**b**) Packaging for fixed location sensing; (**c**) LEDIF block diagram; (**d**) LEDIF packaged inside a 30 (L) × 20 (Dia) cm cylindrical pressure hull for autonomous platform deployment.

The LEDIF, shown in Figure 1, has been described previously in detail [11,12,13]. In this work, its six junctions are fitted with optically enhanced LEDs of 371, 402, 523, 572, 595, 612 nm wavelengths, focused on the analytical volume and oriented at 90° to the main axis of light collection. Absorbance is measured using a broadband (185 to 1100 nm) light source coupled via an optical fiber; a collimation lens illuminates the flow cell at 180° to the light collection system. Turbidity is measured by nephelometry within the flow cell, at each LED wavelength. Flow into the cell is via a pathway that contains two rectangular bends to minimize the entrance of stray light.

Light from the flow cell is observed with a USB4000 spectrometer (Ocean Optics, Dunedin, FL, USA) and the data are recorded with a single-board computer manufactured by Technologic Systems (Fountain Hills, AZ, USA, Model TS-7260-64-128F) running custom software (iLEDLIF). For land-based use in continuous monitoring, a Model SFBP1-G350-01 bilge pump (Seaflo, Xiamen, China) feeds samples into the LEDIF flow cell manifold. An automated compressed air cleaning system constructed with a compressed air cylinder regulated by a double stage pressure regulator connected to a solenoid valve controlled by the iLEDLIF is developed to clean the LEDIF manifold when deployed in situ to discourage fouling and to delay sensor maintenance. When used in the autonomous underwater vehicle (AUV), ram pressure drives samples through the flow cell.

### 2.2. Algae Cultivation

Four genera of algae were cultivated for in vivo laboratory measurements: (a) The green algae (Chlorophyta) group comprised *Ankistrodesmus* (Anki) and *Chlorella* (Chlor); (b) The blue-green algae (Cyanobacteria) group included *Anabaena* (Ana) and *Cylindrospermum* (Cyl); (c) The golden-brown algae (Bacillariophyta, Diatom) group encompassed *Cyclotella* (Cyc); and (d) the red algae (Rhodophyta) group consisted of *Porphyridium* (Porp). The cultivation conditions are summarized in Table 1. The algae stocks were obtained from Carolina Biological Supply Company (Burlington, NC, USA). Model Dual Pro T5HO 39WX2 aquarium lighting (Odyssea, Foshan, China) with time sequences of 12 h light: 12 h dark cycle controlled by a digital timer provides illumination to the culture. For cyanobacteria and red algae, 24 h of continuous light was found to promote quick growth. Diffuser film and cardboard were used to moderate the intensity of light delivered to the culture. The light and temperature were measured with a Model PM100A-S120VC optical meter (Thorlabs, Newton, NJ, USA) and aquarium thermometer, respectively. Aeration to the culture was provided by aquarium air pump (e.g., K-8000, Shiruba, Changhua, Taiwan) connected to an air splitter equipped with flow rate controller. The container, tubing, and laboratory apparatus were sterilized before cultivation.

### 2.3. Cell Counts and Dry Weight

Cell counts of the cultures were performed using a Cellometer^®^ Mini (Nexcelom Bioscience, Lawrence, MA, USA) and/or Bright-Line 3100 hemacytometer (Hausser, Horsham, PA, USA) observed under a Model 44348 PentaView LCD digital microscope (Celestron, Torrance, CA, USA). The dimensions of the algae cell observed under the microscope were scaled using a Model R2L2S1P1resolution target (Thorlabs). For Ana and Cyl, the cultures were sonicated (without rupturing the cells) for 2 min to minimize filamentous clustering for the improvement of counting accuracy.

The dry weight measurements were taken when the cultivated algae reached the maximum cell size except for Anki, Chlor, and Porp where measurements at a different mean cell size were also made. Cultures of known volume and cell counts were filtered with Millipore^®^ glass-fiber filters (Type 5, Lot 3110, Merck Millipore, Sigma-Aldrich, Singapore) instrumented in 47 mm filter holders (Swin-Lok™, Sigma-Aldrich, Singapore). The glass-fiber filter was dried in a Heratherm Oven (Fisher Scientific, Singapore) for more than 24 h at 60 °C before and after the filtration prior to measurement. For Cyc in Alga-Gro^®^ Seawater medium, the sample was centrifuged using a Model 3740 Micro Refrigerated Centrifuge (Kubota, Bunkyo-ku, Tokyo, Japan) at 120,000 rpm and 4 °C for 20 min. The centrifugate (algae) was harvested, filtered, and washed (e.g., to remove salt content) with ammonium bicarbonate [16] obtained from Sigma-Aldrich (CAS 1066-33-7, Product No. 09830) dissolved in Pere Ocean distilled (DI) drinking water before drying and measurement. For Porp in Alga-Gro^®^ Seawater medium, the sample was filtered and washed with DI water or ammonium bicarbonate dissolved in DI water before drying and measurement.

### 2.4. Dissolved Pigments

Biological pigments relevant to the targeted algal groups and humic acid representing potentially interfering dissolved organic matter were measured with the LEDIF. All experiments were performed using laboratory-prepared standards in DI water in amber glass bottles. Phycocyanin (Product No. 52468) and phycoerythrin (CAS 11016-17-4, Product No. 52412) were obtained from Sigma Aldrich, chlorophyll a (CAS 1406-65-1) was from Tokyo Chemical Industry (Chuo-ku, Tokyo, Japan), humic acid (Sodium Salt, tech., CAS 68131-04-4, Lot A0365354) was from Acros Organics (Thermo Fisher, Singapore), and Suwannee River humic acid standard II (Catalog No. 2S101H) from International Humic Substances Society (St Paul, MN, USA). For chlorophyll a, the concentrations reported in this paper are based on the manufacturer’s reported assay of 0.6% plant-derived chlorophyll. For humic acid from Acros Organics, the concentration reported is based on 55% carbon content; the manufacturer’s assay reports carbon content ranging from 45 to 70%. Finally, a multi-spectral and excitation-emission matrix of an aqueous mixture containing several dissolved pigments and humic acid were captured.

For all experiments, the baseline and background signals were corrected with the DI water spectrum used to prepare the laboratory standards. The stock solutions of chlorophyll a and humic acid were filtered with Grade 42 filter paper (GE Healthcare Life Sciences, Pittsburgh, PA, USA), having a nominal pore size of 2.5 µm, to minimize the possible effects of large particle interference (if any) on the fluorescence signal. Fresh solutions were prepared, typically within 2 to 3 h of analysis, and all spectra were collected at room temperature (22 to 25 °C). The integration times for all fluorescence measurements were 10 s. Sensor responses as a function of pigments concentrations were obtained by correlating fluorescence peak intensities recorded in spectra to standards of known concentrations.

### 2.5. In Vivo Measurement

All experiments were performed using laboratory cultivated freshwater and marine algae diluted with DI water (to discourage growth during experiments), Alga-Gro^®^ Seawater Medium, or seawater prepared from Blue Treasure SPS sea salt to the targeted concentration and measured with the LEDIF. Validation runs were performed by preparing two samples, one diluted with Alga-Gro^®^ Freshwater medium and another with DI water to the same concentration and let stand for at least 6 h. The percent differences in fluorescence signals were found to never exceed 2.7 and 4.9%, for phycocyanin and chlorophyll a, respectively. Experimental measurements were performed at different cell concentrations and the baseline and background signals were corrected with the DI water or the seawater spectrum, respectively. Fresh solutions were prepared, typically within 2 to 3 h of analysis from the cultivated stock. All spectra were collected at room temperature (22 to 25 °C). The integration times for all fluorescence measurements were 10 s except for Porp at 5 s.

### 2.6. Extracted Pigment

Fucoxanthin in known cell counts of Cyc culture was extracted by mixing it with equal portion of methanol from Sigma-Aldrich (CAS 67-56-1, Product No. 179337, Lot STBF8801V) for 30 min. The extracted pigment solution was filtered with Sigma-Aldrich Millipore^®^ glass-fiber filters and then diluted to the targeted concentrations for absorbance measurements.

### 2.7. Algae Mixture and Field Sample

Equal volume of known cell counts of Anki, Chlor, Ana and Cyl were mixed to form an algae mixture in the absence and presence of humic acid and the LEDIF was used for fluorescence measurement. The results were compared with the cumulative fluorescence intensities obtained by summing all the fluorescence signals of pure algae samples having the same concentration as the mixture. A field sample collected from a local reservoir in Singapore was measured using the fluorescence mode of the LEDIF at 1 s integration time. Cell counting was performed using the hemacytometer and the cell size was measured with the cellometer. The identification of the dominant genus of algae was observed using microscope.

## 3. Results and Discussion

### 3.1. Qualification of Instrument

#### 3.1.1. Excitation Source Wavelengths

**Figure 2:**
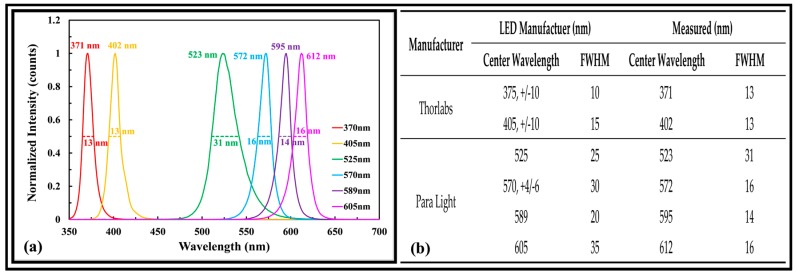
Qualification of LEDIF’s excitation sources: (**a**) Measurement of LED central wavelengths and FWHM bandwidths using LEDIF’s NIST-calibrated spectrometer; (**b**) Comparison of manufacturer’s reported values and LEDIF’s measured values.

The qualification (i.e., characterization) of the LEDIF’s spectrometer has been described by [15]. Figure 2a shows the measured center wavelength and full width at half maximum (FWHM) bandwidth of the six excitation sources instrumented in the LEDIF for this work. The values reported by the LED manufacturers (UV–Thorlabs and VIS–Para Light) are shown in the legend of Figure 2a. In Figure 2b, the LED manufacturer’s reported values are compared with those measured by the LEDIF. The FWHM bandwidths of 375 and 525 nm as measured was somewhat larger than those reported by the manufacturer.

#### 3.1.2. Cell Counts and Dry Weight

**Figure 3:**
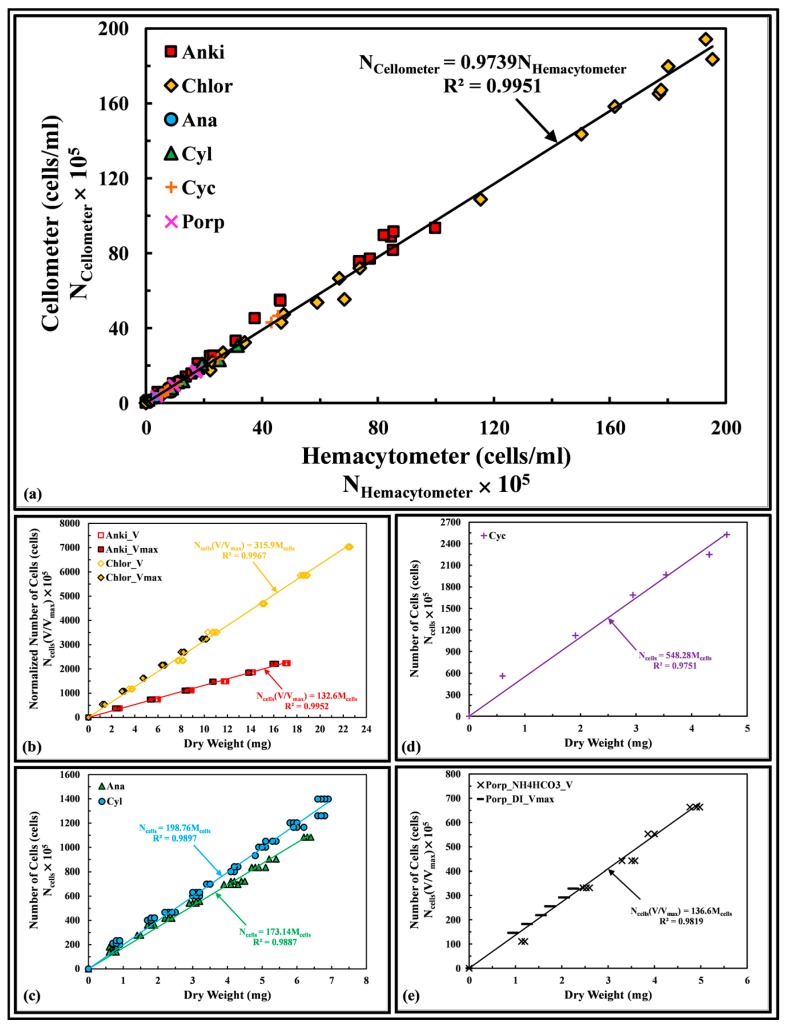
(**a**) Characterization of cell counting instruments. Number of cells-to-dry weight correlations of different genera of (**b**) green; (**c**) cyanobacteria, (**d**) golden-brown; and (**e**) red algae.

Figure 3a compares the cell counts of different samples observed using the cellometer and hemacytometer for the genera of algae used in this work. The gradient of the correlation (0.9739) and the regression coefficient *R*^2^ (0.9951) were close to 1, showing that the results observed by the cellometer match well with the hemacytometer over the range of measurement. Figure 3b–e shows the number of cells measured with the hemacytometer is a linear function of the dry weight of the cells for the genera of algae used in this work. For Anki and Chlor, when two cultivated (i.e., growing and fully grown cells) samples have different cell sizes, the number of cells can be normalized by the cell size volume ratio [mean cell volume (V) over maximum cell volume (V_max_)], and the result is a linear function of the dry weight. The same was observed for Porp and there was no significant difference in using DI water or ammonium bicarbonate (NH_4_HCO_3_) to wash away the salt content [16]. These results provide a mean to correlate the LEDIF signal to the equivalent dry weight of the cells based on the cell counts of the tested algae.

### 3.2. Dissolved Pigments

**Figure 4:**
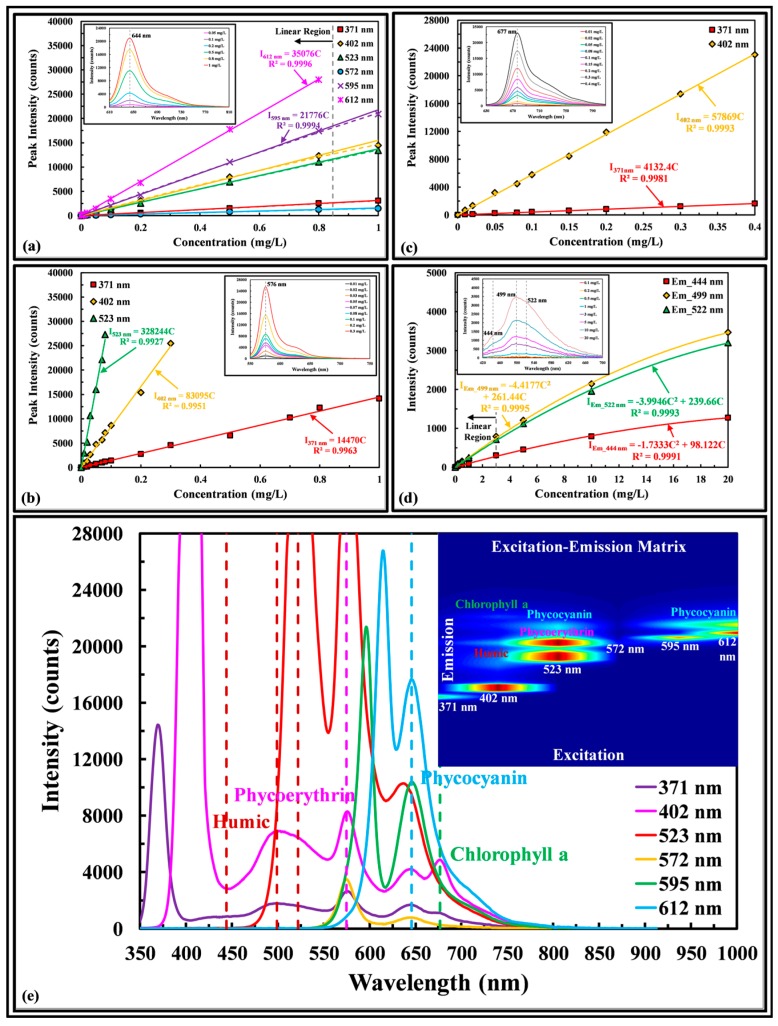
Measurement of aqueous (**a**) phycocyanin, (**b**) phycoerythrin, (**c**) chlorophyll a fluorescence peak intensity as a function of concentration at different excitation wavelengths. Insert graphs show the emission spectra excited at (**a**) 595 nm, (**b**) and (**c**) 402 nm; (**d**) Measurement of Suwannee River humic acid fluorescence peak intensity as a function of concentration at three different emission wavelengths. Insert graph shows emission spectrum excited at 371 nm; (**e**) Emission spectrum and excitation-emission matrix of a complex lab mixture.

Figure 4a shows the fluorescence spectra and fluorescence peak intensity of phycocyanin as a function of concentration at six excitation wavelengths. The fluorescence peak intensity is a linear function of concentration up to 0.847 mg/L, with a small deviation of 3.9 to 6.8% from linearity for higher concentrations up to 1 mg/L, which may be attributed to inner filtering, as the LEDIF has an optical pathlength of 9 mm (as opposed to 5 mm of a standard cuvette) to accommodate the physical dimensions of the six LED excitation systems. The fluorescence peak of phycocyanin reported by the LEDIF was 644 nm, having percent difference of 0.16% with the value 643 nm reported by the manufacturer (Sigma-Aldrich).

Figure 4b shows the fluorescence spectra and fluorescence peak intensity of phycoerythrin as a function of concentration at three excitation wavelengths. The fluorescence peak intensity is a linear function of concentration up to 1 mg/L and the fluorescence peak was 576 nm having percent difference of 0.17% with the value 575 nm reported by the manufacturer (Sigma-Aldrich). Figure 4c shows the fluorescence spectra and fluorescence peak intensity of chlorophyll a as a function of concentration at two excitation wavelengths. The florescence peak intensity is a linear function of concentration up to 0.4 mg/L and the fluorescence peak was 677 nm, agreeing well with values from the literature. Figure 4d shows the fluorescence spectra and three fluorescence peak intensities of Suwannee River humic acid as a function of concentration excited by 371 nm wavelength. The fluorescence peak intensity is a linear function of concentration up to 3 mg/L and the fluorescence peaks were 444, 499, and 522 nm. At concentration higher than 3 mg/L, the inner filtering compensation procedure of [15] can be adapted to correct for non-linearity. No shift of fluorescence peak with excitation wavelengths is observed for all dissolved pigments. Figure 4e shows the emission spectrum and excitation-emission matrix (EEM) of an aqueous solution containing 0.1 mg/L chlorophyll a, 0.6 mg/L phycocyanin, 0.1 mg/L phycoerythrin, and 5 mg/L humic acid; representing biological pigments found in green algae, cyanobacteria, red algae, and dissolved organic matters. Each pigment can be clearly identified by inspection. The fluorescence peak intensities were 677, 646, and 575 nm respectively for the three biological pigments and 522, 499 and 422 nm for the three emission peaks of humic acid. No shift of fluorescence peaks with excitation wavelengths are observed for the mixture except for a 9.6 nm blue shift of phycocyanin fluorescence peak excited at 523 nm.

### 3.3. In Vivo Measurement

Each data point in the graphs is an average of five measurements and each experiments were repeated at least twice on a different days using freshly prepared samples.

#### 3.3.1. Green Algae

**Figure 5:**
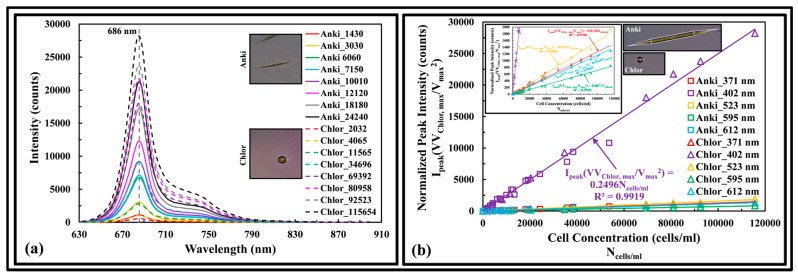
(**a**) Fluorescence spectra of Anki and Chlor of different cell concentrations excited at 402 nm wavelength. Legend: genus of algae_cells per mL; (**b**) Normalized peak intensity of Anki and Chlor as a function of cell concentration. Legend: genus of algae_excitation wavelength.

Figure 5a shows the fluorescence spectra of Anki and Chlor excited at 402 nm wavelength. The dry weight of the cells ranged from 0.145 to 4.05 mg/L and 0.0643 to 3.66 mg/L, respectively. The fluorescence peaks at 686 nm, a red shift of 9 nm from the measured dissolved chlorophyll a pigment. By taking the geometric cell shape of the Anki to be cylindrical with conical ends (insert graph of Figure 5b) and the Chlor to be spherical, the mean volume of the cells was computed as 1902 and 524 µm^3^, based on mean cell size of 5.697 (Dia) × 116.6 (L) and 10 µm, respectively. Figure 5b shows that by normalizing the fluorescence peak intensity with the targeted cell size volume ratio [mean cell volume (V) over maximum cell volume (V_max_)] and the ratio of Chlor maximum cell volume (V_Chlor, max_) over targeted cell volume (V), and plot the data as a function of cell concentration, a linear calibration is obtained for each excitation wavelength.

#### 3.3.2. Cyanobacteria (Blue-Green Algae)

**Figure 6:**
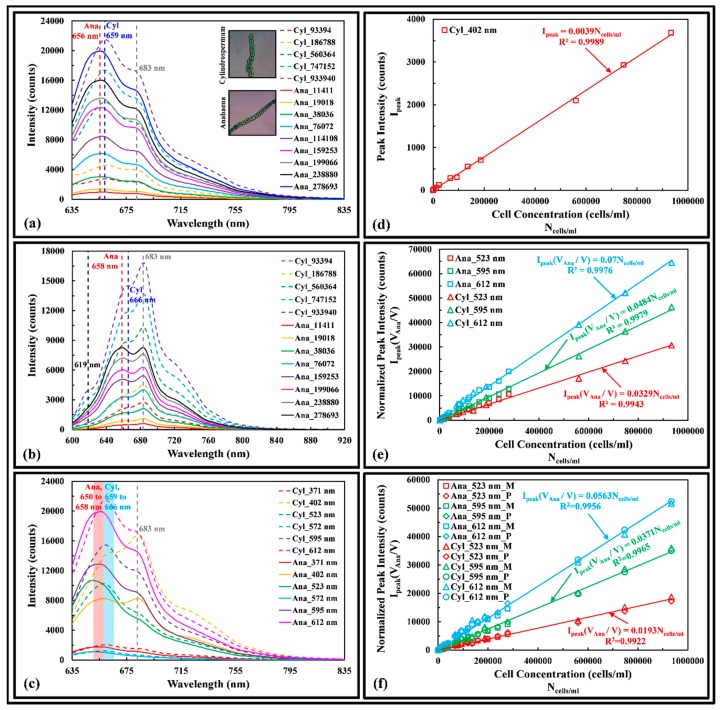
Fluorescence spectra of Ana and Cyl of different cell concentrations excited at (**a**) 612 nm and (**b**) 402 nm wavelength. Legend: genus of algae_cells per mL; (**c**) Fluorescence spectra of Ana and Cyl excited at different wavelengths; (**d**) 619 nm emission peak of Cyl as a function of cell concentration; Normalized (**e**) phycocyanin and (**f**) measured and predicted chlorophyll a peak intensities of Ana and Cyl as a function of cell concentration. Legend: genus of algae_excitation wavelength_M is measured and P is predicted data.

Figure 6a shows the fluorescence spectra of Ana and Cyl excited at 612 nm wavelength. The dry weight of the cells ranged from 0.0758 to 16.1 mg/L and 0.114 to 47.0 mg/L, respectively. The phycocyanin fluorescence of Ana and Cyl peak at 656 and 659 nm, a red shift of 12 and 15 nm relative to dissolved phycocyanin. Figure 6b shows the fluorescence spectra of Ana and Cyl excited at 402 nm wavelength. Contrary to 612 nm excitation wavelength, the ratio of phycocyanin to chlorophyll a intensity increases as the cell concentration of Ana increases. The phycocyanin fluorescence of Ana and Cyl peak at 658 and 666 nm, a longer red shift of 14 and 22 nm from the dissolved phycocyanin pigment, respectively. The Cyl has an emission peak at 619 nm that was not observed in the Ana fluorescence spectra; which may serve as a distinctive signature to distinguish between Cyl and Ana.

Figure 6c shows the fluorescence spectra of Ana and Cyl excited at different wavelengths. The phycocyanin fluorescence of Ana and Cyl peak between 650 to 658 nm (red shaded region) and 659 to 666 nm (blue shaded region) respectively, when excited by different wavelengths. The chlorophyll a fluorescence peaks at 683 nm (Figure 6a–c) for both Ana and Cyl, a red shift of 6 nm from the measured dissolved chlorophyll a pigments but it is independent of the excitation wavelengths used in this work. Figure 6d shows the 619 nm emission peak of Cyl is a linear function of cell concentration. By taking the geometric cell shape of the Ana and Cyl to be ellipsoidal, the mean volume of the beaded cells were computed as 446 µm^3^ and 149 µm^3^, based on mean cell size of 11.48 × 8.61 × 8.61 and 8.61 × 5.74 × 5.74 µm, respectively. Figure 6e shows that by normalizing the fluorescence peak intensity of Ana and Cyl with the cell volume ratio [Ana cell volume (V_Ana_) over targeted cell volume (V)] and plot the data as a function of cell concentration, a linear calibration is obtained when excited at 523, 595 and 612 nm. By taking the ratio of the fluorescence peak intensity of phycocyanin over chlorophyll a at each cell concentration, a scaling factor ($S_{\alpha }$) can be derived for each excitation wavelength, allowing the prediction of unknown chlorophyll a peak intensity from known phycocyanin peak intensity of Ana and Cyl. The scaling factor can be described by:

(1) $$S_{\alpha }=\frac{I_{phycya}}{I_{chlo\;a}}=3\frac{\lambda_{em\left(phycya\right)}}{\lambda_{ex}}-2$$

where $I_{phycya}$ and $I_{chlo\;a}$ are the phycocyanin and chlorophyll a fluorescence peak intensities, respectively, $\lambda_{em\left(phycya\right)}$ is the emission wavelength of phycocyanin, and $\lambda_{ex}$ is the excitation wavelength. Equation 1 may be used to quantify the cell concentration of green algae in the presence of cyanobacteria. Figure 6f shows both the measured and predicted chlorophyll a peak intensity of Ana and Cyl is linearly proportional to cell concentration when excited at 523, 595, and 612 nm.

#### 3.3.3. Red Algae

**Figure 7:**
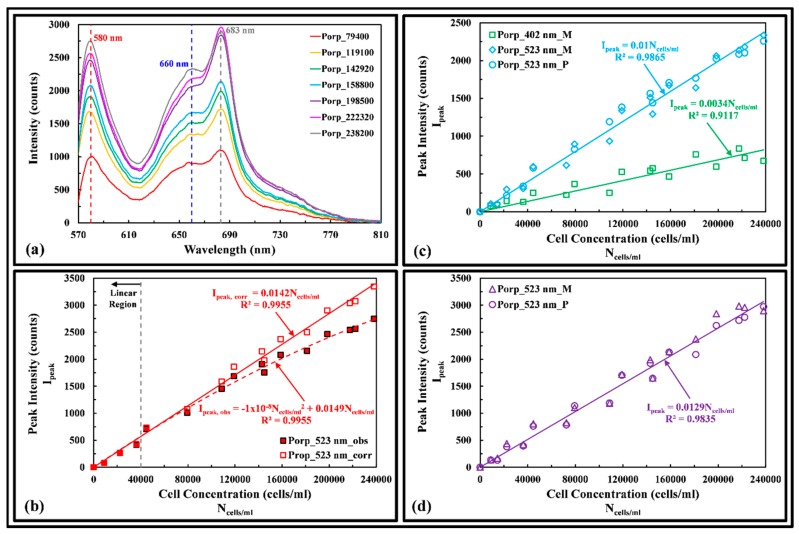
(**a**) Fluorescence spectra of Porp of different cell concentrations (cells/mL) excited at 523 nm; (**b**) Observed (obs) and inner-filtering corrected (corr) phycoerythrin fluorescence peak intensities of Prop as a function of cell concentration excited at 523 nm; (**c**) Measured (M) and predicted (P) phycocyanin peak intensity of Porp as a function of cell concentration excited at 402 nm and 523 nm; (**d**) Measured (M) and predicted (P) chlorophyll a peak intensities of Porp as a function of cell concentration excited at 523 nm.

Figure 7a shows the fluorescence spectra of Porp excited at 523 nm wavelength. The dry weight of the cells ranged from 0.65 to 17.44 mg/L. The phycoerythrin and chlorophyll a fluorescence peak at 580 and 683 nm, a red shift of 4 and 6 nm from the measured dissolved pigments, respectively. It was observed that chlorophyll a fluorescence from 198,500 to 238,200 cells/mL begins to overlap one another, when inner filtering becomes prominent. The phycocyanin fluorescence peak at 660 nm, a red shift of 16 nm from the measured dissolved pigment. By taking the geometric cell shape of the Porp as spherical, the mean volume of the cells was computed as 268 µm^3^, based on mean cell size of 8 µm. Figure 7b shows both the observed and the inner-filtering corrected phycoerythrin fluores-cence peak measurements of Prop. Absorbance was tested and found to be approximately linear up to 264,000 cells/mL. The absorptivity for 523 nm excitation and the 580 nm emission wavelengths was computed as 2.0719 × 10^−7^ and 1.9743 × 10^−7^ (cells/mL)^−1^·cm^−1^, with excitation and emission optical path lengths of 8.7 and 9 mm, respectively {Beer-Lambert [17] equation, A= εlC was used, where A is absorbance (absorbance unit), ε represents absorptivity, l denotes optical path length, and C is the concentration}. The inner-filtering effect was then corrected {[15] equation, $I_{corr}=I_{obs}antilog_{10}[{\left(OD_{ex}\right)}_{l,corr}+{\left(OD_{em}\right)}_{l,corr}]$ was used, where $I_{corr}$ and $I_{obs}$ are the corrected and observed fluorescence peak intensities, respectively, $antilog_{10}$ denotes antilog base 10, and ${\left(OD_{ex}\right)}_{l,corr}$ and ${\left(OD_{em}\right)}_{l,corr}$ represent the path-length-corrected excitation and emission optical densities, respectively}. By taking the ratio of the fluorescence peak intensity of phycoerythrin over phycocyanin at each cell concentration, a scaling factor ($S_{\beta }$) can be derived, allowing the prediction of unknown phycocyanin peak intensity from known phycoerythrin peak intensity of Porp. The scaling factor can be described by:

(2) $$S_{\beta }=\frac{I_{phyery}}{I_{phycya}}=2\frac{\lambda_{em\left(phyery\right)}}{\lambda_{ex}}-1$$

where $I_{phyery}$ and $I_{phycya}$ are the phycoerythrin and phycocyanin fluorescence peak intensities, respectively, $\lambda_{em\left(phyery\right)}$ is the emission wavelength of phycoerythrin, and $\lambda_{ex}$ is the excitation wavelength. Figure 7c shows the phycocyanin fluorescence peak of Prop is a linear function of cell concentration when excited at 402 and 523 nm. Equation (2) may be used to quantify the cell concentration of cyanobacteria in the presence of red algae. The same can be done to the fluorescence peak of phycocyanin and chlorophyll a and the scaling factor ($S_{\gamma }$) can be described by:

(3) $$S_{\gamma }=\frac{I_{phycya}}{I_{chlo\;a}}=3\left[\frac{\lambda_{em\left(phycya\right)}}{\lambda_{ex}}-1\right]=S_{\alpha }-1$$

where $I_{chlo\;a}$ is the chlorophyll a fluorescence peak intensity and $\lambda_{em\left(phycya\right)}$ is the emission wavelength of phycocyanin. Equation (3) may be used in conjunction with Equation (1) to quantify the cell concentration of green algae in the presence of cyanobacteria and red algae. Figure 7d shows both the measured and predicted chlorophyll a peak intensity of Porp is linearly proportional to cell concentration when excited at 523 nm. By combining Equations (2) and (3), the ratio ($S_{\delta }$) of the fluorescence peak intensity of phycoerythrin over chlorophyll a can be described by:

(4) $$S_{\delta }=\frac{I_{phyery}}{I_{chlo\;a}}=S_{\beta }S_{\gamma }=3\left[2\frac{\lambda_{em\left(phyery\right)}}{\lambda_{ex}}-1\right]\left[\frac{\lambda_{em\left(phycya\right)}}{\lambda_{ex}}-1\right]$$

Using Equation (4), for 523 nm excitation, $S_{\delta }$ is computed as 0.9568. By taking the average value of $I_{phyery}/I_{chlo\;a}$ at each cell concentration when excited at 523 nm, the average value (0.9042) is 5.5% lower than the computed value.

#### 3.3.4. Golden-Brown Algae

**Figure 8:**
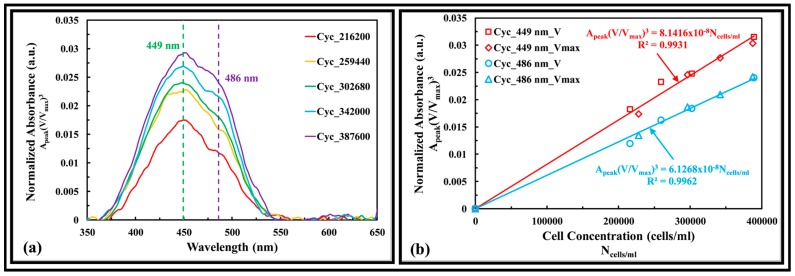
(**a**) Absorbance spectra of Cyc of different cell concentrations (cells/mL). Legend: genus of algae_cells per mL; (**b**) Fucoxanthin absorbance peaks of Cyc as a function of cell concentration. Legend: genus of algae_absorbance peak wavelength_V is growing and V_max_ is fully grown algae.

Figure 8a shows the absorbance spectra of Cyc. The dry weight of the cells ranged from 3.94 to 7.10 mg/L. The absorbance peaks at 449 and 486 nm. By taking the geometric cell shape of the Cyc to be a short cylinder, the mean volume of the cells was computed as 572 µm^3^, based on mean cell size of 10.3 (Dia) × 6.8 (L). Figure 8b shows that by normalizing the absorbance peak with the targeted cell size volume of two cultivated (i.e., growing and fully grown) samples having different cell size, and plot that as a function of cell concentration, a linear calibration is obtained for absorbance peak of 449 and 486 nm. Based on linear regression fitting to the Beer-Lambert [17] curve (*A* = *εlC*) and the optical path length of the LEDIF, the absorptivities were calculated as 2.05 × 10^−8^ and 1.54 × 10^−8^ (cells/mL)^−1^·cm^−1^, absorbance peak of 449 and 486 nm, respectively.

#### 3.3.5. Algae Mixture and Field Samples

**Figure 9:**
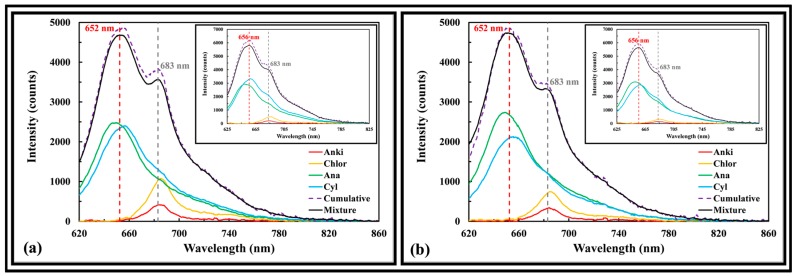
Fluorescence spectra of (**a**) Anki, Chlor, Ana, and Cyl mixture and (**b**) Anki, Chlor, Ana, Cyl, and 5 mg/L humic acid mixture excited at 523 and 595 nm (insert graph).

Figure 9a shows the fluorescence intensity of an Anki, Chlor, Ana, and Cyl mixture matches the cumulative fluorescence intensities of pure algae samples having the same concentration as the mixture. The percent difference at the phycocyanin and chlorophyll a peaks were 4.1 and 7.5% when excited at 523 nm. When excited at 595 nm, the percent difference was 7.3 and 6.5%, respectively. Figure 9b shows the same can be observed in the presence of 5 mg/L humic acid (e.g., background signal from organic constituents in water bodies), with a percent difference of 2.7 and 6.3% (excited at 523 nm) and 5.4 and 9.9% (excited at 595 nm) at the phycocyanin and chlorophyll a peaks, pointing that correction may not be necessary when background signal due to humic acid is modest. The results show that it is feasible to use the fluorescence signal of a mixture to classify and quantify the group of algae presence in a water sample.

**Figure 10:**
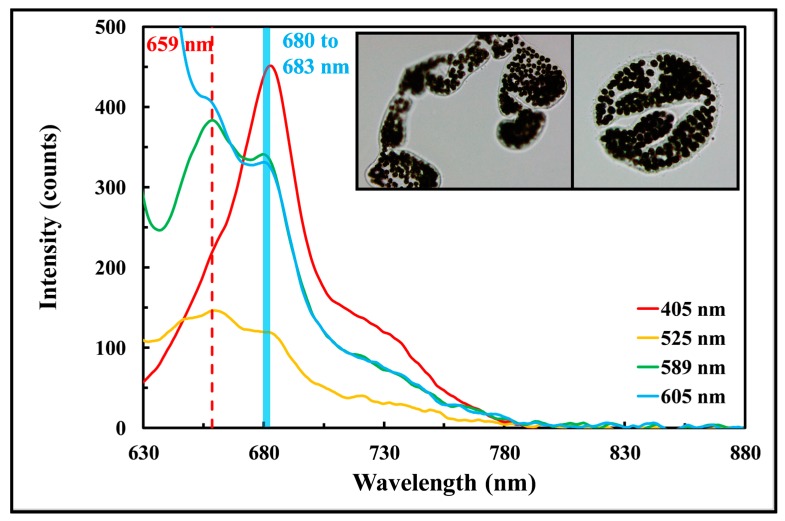
Fluorescence spectra of a field sample dominated by *Microcystis* (insert graph) observed in a local reservoir in Singapore.

Figure 10 shows the fluorescence spectra of a field sample collected on 28 February 2017 from a local reservoir in Singapore at 1 second integration time. The phycocyanin and chlorophyll a peak at 659 and between 680 to 683 nm (blue shaded region), respectively; which matches with the peak wavelengths observed in the laboratory cultivated Ana and Cyl. The dominated genus of algae is *Microcystis* (insert graph), a group of toxic cyanobacteria that is commonly observed in eutrophic fresh water. The cell counts measured with the hemacytometer was 4.56 × 10^5^ cells/mL and the cell size reported by the cellometer was 10.28 µm. The cell volume ratio of Ana and *Microcystis* was computed as 0.78.

The absorption of phycocyanin in cyanobacteria peaks close to 612 nm. By applying calibration correlations of phycocyanin (Figure 6e) and chlorophyll a (Figure 6f) excited at 612 nm to the fluorescence signal of *Microcystis*, the cell concentrations were computed as 4.54 × 10^5^ and 4.62 × 10^5^ cells/mL, a percent difference of 0.44 and 1.3% from the hemacytometer measurement. The results show that it is possible to use the LEDIF with the calibration correlations to quantify the cell concentration of a different cyanobacteria, although further study will be required to characterize this finding.

## 4. Conclusions

The results described illustrate the LEDIF’s capabilities in measuring both the in vivo and in vitro biological pigments present in four different groups of algal, and suggest that using them for algae classification and quantification are possible. These capabilities can be readily implemented to different platforms of the LEDIF to achieve observations covering both space and time. The LEDIF is also capable of measuring other biological pigments such as chlorophyll b in Chlorophytes and Euglenophytes that were not presented in this paper. Matrix effects in these measurements were not investigated, which, could be important and made possible by adapting the compensation techniques of [15]. Finally, a spectrum separation algorithm can be applied to improve the quantification of algae mixtures.

## Figures and Tables

**Table 1:** Algae cultivation conditions.

Group	Genus	Culture Medium	Light (Foot-Candles)	Optimum Temperature (°C)
Green	*Ankistrodesmus Chlorella*	Alga-Gro^®^ Freshwater	200 to 400	22
Blue-Green (Cyanobacteria)	*Anabaena Cylindrospermum*	Alga-Gro^®^ Freshwater	50 to 100	
Golden-Brown	*Cyclotella*	Alga-Gro^®^ Seawater	200 to 400	
Red	*Porphyridium*	Alga-Gro^®^ Seawater	50 to 100	
